# Psychometric evaluation of the Values Inventory: structural validity, internal consistency, and the integration with sleep value in a U.S. adult online sample

**DOI:** 10.3389/frsle.2026.1800391

**Published:** 2026-05-22

**Authors:** Dustin Sherriff, Abby Woolley, Brandon Pattillo, Chongming Yang, Kara M. Duraccio, Daniel B. Kay

**Affiliations:** 1 Department of Psychology, Brigham Young University, Provo, UT, United States; 2 Department of Cell Biology & Physiology, Brigham Young University, Provo, UT, United States; 3 FHSS Research Support Center, Brigham Young University, Provo, UT, United States

**Keywords:** life values, sleep profiles, sleep valuation, sleep value, terminal values

## Abstract

Personal values shape health behaviors, yet the role of sleep value within broader value systems remains unknown. This study aimed to validate the Values Inventory, a novel measure assessing individual life values, and examine how sleep value fits into broader these value orientations. An online sample of U.S. adults (*N* = 455) completed the Values Inventory and the Sleep Valuation Item Bank 2.0 (SVIB-2.0), along with demographic and sleep-related surveys. Exploratory factor mixture modeling and confirmatory factor analysis supported a five-factor structure of the Values Inventory: Health/Wellbeing, Fundamental Human Values, Social Status, Personal Accomplishment/Global Advancement, and Community/Belonging. Internal consistency was high across factors (ω ≥ 0.88). Structural equation modeling revealed significant associations between demographic characteristics and value endorsements. Sleep health, along with mental and physical health, formed the core of the Health/Wellbeing factor, supporting the common belief that sleep is a pillar of health. Age was significantly associated with higher valuing of Health/Wellbeing and Fundamental Human Values. Multivariate analysis of variances comparing Values Inventory factors across previously established sleep value profiles (Unconcerned, Appreciative, Devalue, Ambivalent Priority, and Concerned) revealed distinct value patterns. The Appreciative profile showed the highest valuation of Health/Wellbeing. The Ambivalent Priority profile showed the highest valuation of Social Status and Community/Belonging. Findings support the Values Inventory as a psychometrically sound tool for assessing individual value systems and highlight the complex role of sleep. These results suggest the need for future research determining whether aligning sleep health interventions with individuals' broader values may enhance effectiveness and relevance.

## Introduction

Sleep value, defined as the relative worth an individual places on their sleep (Nielson et al., 2021), plays a vital role in shaping sleep health. Sleep health is a multidimensional concept that adapts to individual, social, and environmental demands, directly impacting both physical and mental health (Buysse, 2014). Poor sleep has been linked to an increased risk of cardiovascular disease, hypertension, diabetes, and other physical and psychological challenges (Knutson et al., 2011; Laugsand et al., 2011; Vgontzas et al., 2009; Wells and Vaughn, 2012). Despite its critical role, more than a third of American adults do not get enough sleep on a regular basis (CDC, 2016). Moreover, sleep remains undervalued in healthcare settings, as family medicine clinics do not screen for sleep issues as frequently as other behavioral and lifestyle factors when establishing patient health histories (Sorscher, 2008). A better understanding of how sleep value relates to individuals' broader value systems could help identify how individuals engage in sleep health behaviors, offering a novel target for interventions aimed at improving sleep health outcomes.

The most recent research on sleep value has focused on how it can be measured and conceptualized. Sherriff et al. (2025a)) identified five factors of sleep value: wanting, prioritizing, devaluing, appreciating, and preferring sleep. Using these five factors, they also identified five distinct sleep valuation profiles: Unconcerned, characterized by low engagement and indifference toward sleep; Appreciative, reflecting a positive but non-urgent valuation of sleep; Devalue, marked by negative attitudes toward sleep despite engaging in it; Ambivalent Priority, defined by a strong desire and prioritization of sleep coupled with conflicting devaluing beliefs; and Concerned, characterized by high valuation and prioritization of sleep, likely in response to insufficient sleep (Sherriff et al., 2025a). Together, the sleep value factors and profiles derived from the SVIB 2.0 suggest that sleep value is not binary but instead exists along a nuanced spectrum that manifests in predictable subtypes based on individual differences in demographic, sleep, and health characteristics.

Sleep value is a dimension of an individual's value system but has not been integrated into prior frameworks of human values. Schwartz's theory of basic human values identifies fundamental guiding principles such as security, hedonism, and achievement, which influence long-term behaviors and priorities (Schwartz, 2012; Russo et al., 2022). Similarly, Milton Rokeach published a list of 36 values reflecting desirable end-states of existence along with the modes of behavior to achieve those states (Rokeach, 1979). These values include 18 terminal goals (such as happiness and freedom) that illustrate how values inform behavior. These values, which we define as life values, while often enduring, are not static, and evolve over time due to life circumstances such as aging, parenthood, and career progression (Ignjatović et al., 2025; Rokeach, 1974). For instance, military professionals increasingly prioritize sleep as they advance in their careers, recognizing its importance for performance and well-being (Petrofsky et al., 2023). We extended Rokeach's 18 life values framework to include sleep value, providing a means to examine how sleep fits within individuals' broader value systems. This integration will offer insight into how people prioritize an activity that occupies nearly one-third of their lives.

Despite widespread acknowledgment of sleep's importance in society, individual behaviors often do not align with their stated values of sleep. A study conducted by the Korean Sleep Research Society on sleep prioritization vs. practice found that 70.7% of participants ranked sleep among their top three daily activities, yet only 35.7% and 38.1% chose to sleep when it conflicted with work and family responsibilities, respectively (Huang et al., 2023). This discrepancy highlights the complexity between perceived importance and actual behaviors, suggesting that other values may take precedence over sleep health. The misalignment of values and behavior in relation to sleep health is poorly understood. While there has been research on barriers to sleep hygiene and sleep attitudes (Hedin et al., 2020; Ruggiero et al., 2019), sleep value is severely understudied. There is currently no validated tool for measuring core values that includes sleep value, making it difficult to understand the place of sleep health in broader value systems.

To address this gap in the literature, we developed the Values Inventory, a novel questionnaire that aims to measure what individuals value the most in their lives including questions related to health, social status, belonging, and other commonly held values. The items were originally adapted from Rokeach's (1979)) list of 18 terminal health values (i.e., life values) and included updated items to capture modern values. First, this study aimed to validate the Values Inventory including determining its internal consistency, latent structure, and how sleep health value fits into the overall structure of individuals' value system. The seconds aim was to determine how the value factors of the Values Inventory relate to demographic variables. The third aim was to determine how the Values Inventory relates to the Sleep Value Item Bank 2.0 and its sleep value profiles (Kay et al., 2023; Nielson et al., 2021; Sherriff et al., 2025a).

## Methods

These data were collected as part of the Sleep Resilience and Variance in Sleep Valuation (SRVIV) Study (Sherriff et al., 2025b) which aimed to validate the Values Inventory and study sleep resilience. Comprehensive details of the methods in the parent study have been previously reported (Sherriff et al., 2025b). Below we summarize the aspects relevant to the current analyses.

### Participants and recruitment

Participants were recruited by a Qualtrics team and were asked to complete an anonymous online survey. An initial recruitment goal of 500 participants was sought, consistent with conventional recommendations of at least 10 participants per item for factor analytic approaches (Kline, 2014). Although a formal power analysis was not conducted, this sample size was deemed suitable based on the sample size of other studies that utilize factor analysis and structural equation modeling (Ravyts and Dzierzewski, 2024).

A sample of 508 adult participants living in the continental United States were recruited via Qualtrics. Responses were screened for validity using several procedures, including automated bot detection (reCAPTCHA), prevention of duplicate entries, restrictions on web indexing, and exclusion of participants who completed the survey in less time than determined to be feasible (<420 s, based on pilot testing) prior to receiving the final dataset. Further exclusions were made for contradictory, nonsensical, or invariant responding. After exclusions, 455 valid responses were retained. All participants completed the entire survey, and there was no missing data to account for. Participants ranged in age from 18 to 85 years (*M* = 45 ± 17), and the sample was 53% female, 46% male, and 1% non-binary. Racial and ethnic composition was 82% White, 13% Black/African American, 5% Hispanic/Latino/Latina, and half of the participants were married (see Table 1).

**Table 1:** Demographic features of the analysis sample (*N* = 455).

Sample characteristic	Sample metrics
Gender	
Female	242 (53%)
Male	209 (46%)
Non-Binary	4 (1%)
Age	45.4 (16.7)
Race^a^	
White	372 (82%)
Black/African American	58 (13%)
Hispanic/Latino/Latina	23 (5%)
American Indian/Alaska Native	14 (3%)
Asian/Asian American	11 (2%)
Mixed	4 (1%)
Other	2 (0.4%)
Education	
Less than a high school diploma	17 (4%)
High school degree or equivalent	98 (22%)
Some college, no degree	110 (24%)
Associate's degree	46 (10%)
Bachelor's degree	91 (20%)
Master's degree	77 (17%)
Advanced Professional or Doctorate Degree	16 (4%)
Dependents	1.5 (2.1)
Household income	
Less than $10,000	51 (11%)
$10,000– $40,000	145 (32%)
$40,001–$90,000	119 (26%)
$90,001–$190,000	98 (22%)
>$190,000	42 (9%)
Marital status^a^	
Married	226 (50%)
Single	100 (22%)
Divorced	50 (11%)
In a committed relationship	46 (10%)
Widowed	20 (4%)
Never married	12 (3%)
Separated	8 (2%)
Cohabitating	5 (1%)
Self-reported mental health^b^	73.1 (25.8)
Self-reported physical health^b^	70.6 (22.3)

*n (%)* and *M* (SD).

^a^indicates that participants could select multiple options.

^b^indicates that mental and physical health were rated on a scale from 0 to 100.

Table adapted from Sherriff et al. (2026)).

### Procedure

All participants provided electronic informed consent prior to completing the survey. Data collection occurred between July 13 and August 9, 2023. The study was reviewed and approved by the Brigham Young University Institutional Review Board (IRB #2023-146) and conducted in accordance with the Declaration of Helsinki. Upon completion, participants received compensation distributed by the Qualtrics team at an estimated rate of $5.23 per hour (approximately $2.18 for the 25-min survey); however, exact payment details were not disclosed to the researchers.

### Measures

Participants completed a demographic questionnaire that included age, gender (male, female, non-binary), race and ethnicity (White, Black/African American, Hispanic/Latino/Latina, American Indian/Alaska Native, Asian/Asian American, Mixed, Other), marital status (Married, Single, Divorced, In a committed relationship, Widowed, Never married, Separated), number of dependents, education level (Less than a high school diploma, High school degree or equivalent, Some college no degree, Associate's degree, Bachelor's degree, Master's degree, Advanced Professional or Doctorate Degree), income (Less than $10,000, $10,000–$40,000, $40,001–$90,000, $90,001–$190,000, >$190,000), and self-rated mental and physical health (rated on a scale from 0 to 100).

### Values inventory

The Values Inventory is a novel 38-item measure developed to assess life values, which are the ultimate life outcomes that a person strives for, including sleep health. Item development began with Rokeach's (1974)) framework of terminal values, which we call life values. As Rokeach's work reflects societal norms nearly a half-century old, values were revised to capture more contemporary values. This was done by conducting web searches and in-lab brainstorming activities to generate lists of common values, as well as reviewing lists of values in common behavioral activation activities. Items were iteratively revised based on pilot feedback from research assistants at Brigham Young University. Study participants rated the Values Inventory's 38 items on a five-point scale ranging from “Not at all valuable” to “Of utmost value,” and then rank-ordered the values they endorsed as “Of utmost value” into their top 10 values.

### Sleep value item bank 2.0

The SVIB-2.0 is a 60-item instrument that measures multiple dimensions of sleep value, including sleep wanting, prioritizing, devaluing, appreciating, and preferring (Kay et al., 2023; Sherriff et al., 2025a; Nielson et al., 2021). The first iteration of the SVIB, investigated by Nielson et al., demonstrated good internal consistency (α = 0.92) and evidence of concurrent validity, with sleep valuation correlating significantly with metrics of daytime sleepiness, sleep quality, depression, anxiety, and self-rated physical and mental health. The second and current iteration of the SVIB 2.0 has been updated to remove items with poor factor loadings and improve psychometric properties. In this validation study, the reliability measured by omega (based on factor loadings) ranged from 0.88 to 0.95, suggesting good internal consistency (Sherriff et al., 2025a; McNeish, 2018).

## Data analysis

Data cleaning methods were previously reported and highlighted above (Sherriff et al., 2025b). For the current analyses, demographic variables were either dummy-coded or treated as ordinal, depending on their nature. Binary variables (including sex, race, and marital status) were dummy-coded such that a value of 1 reflected the presence of the characteristic (e.g., 1 = female), and 0 indicated its absence (e.g., 0 = male). Education and income were coded as ordinal variables, with education ranging from 0 (less than a high school diploma) to 6 (professional or doctoral degree), and income ranging from 0 (under $10,000) to 4 (over $190,000). The number of dependents represented a count of individuals financially reliant on the participant.

To establish the latent structure of the Values Inventory, we conducted exploratory factor mixture modeling (EFMM) exploring two latent classes and 1–5 factors to examine potential subgroup structure within the dataset. Model fit was evaluated using Akaike Information Criterion (AIC), Bayesian Information Criterion (BIC), sample-size adjusted BIC (ABIC), and entropy. Following the EFMM, the retained five-factor structure was evaluated using confirmatory factor analysis (CFA), with residual covariances allowed among conceptually similar items. Next, a structural equation model (SEM) was conducted regressing the five Values Inventory latent factors identified in the CFA on demographic covariates to examine associations with demographic characteristics. Model fit for both the CFA and SEM were evaluated using the chi-square test of model fit, Comparative Fit Index (CFI), Tucker–Lewis Index (TLI), Root Mean Square Error of Approximation (RMSEA), and Standardized Root Mean Square Residual (SRMR) (McNeish, 2018). Analyses were performed with Mplus, Version 8.11 (Muthén and Muthén, 1998-2023), using weighted least squares mean and variance adjusted estimation (WLSMV) estimator with a probit link function for ordinal indicators and theta parameterization for categorical variables.

Following the SEM, multivariate differences in the five Values Inventory latent factors were examined using previously saved factors scores from the CFA across the previously identified Sleep Value Profiles from the SVIB 2.0: Unconcerned, Appreciative, Devalue, Ambivalent Priority, and Concerned. Deviation contrast coding was applied to compare each Sleep Value Profile group with the unconcerned group as the reference category (with zero means on the latent scales). This approach allowed us to assess whether each group significantly differed from the average of all groups. This post hoc multivariate analysis of variance (MANOVA) was performed using IBM SPSS Statistics (Version 29.0.1.0).

## Results

Fit indices for the EFMM factor solutions are presented in Table 2. A two-class model was examined, and model fit improved as the number of factors increased from one to five, as indicated by decreasing AIC and ABIC values and increasing entropy. The four-factor model failed to converge. Although the five-factor model demonstrated the lowest AIC and ABIC values and highest entropy (0.936), the BIC value increased slightly relative to the three-factor solution. However, inspection of the factor loadings indicated that the five-factor solution provided the clearest and most interpretable structure, with items demonstrating strong primary loadings and minimal cross-loadings across the two latent classes. The factor structure was largely consistent across the two latent classes, suggesting that differences between classes reflected variation in endorsement levels rather than differences in measurement structure.

**Table 2:** Fit indices for exploratory factor mixture models of the values inventory (two-class solutions).

Number of factors	AIC	BIC	ABIC	Entropy
1	39,154.3	40,724.1	39,515.0	0
2	36,465.5	38,340.2	36,896.2	0.905
3	35,691.8	37,863.2	36,190.7	0.910
4	—	—	—	—
5	35,526.5	38,266.5	36,156.0	0.936

AIC, akaike information criterion; BIC, Bayesian information criterion; ABIC, sample-size adjusted Bayesian information criterion. Lower values indicate better model fit. The four-factor model failed to converge.

Based on these results, a five-factor structure was retained and evaluated using CFA. The five-factor CFA model of the Values Inventory demonstrated a strong fit (χ^2^_(127)_ = 761.35, *p* < 0.001, CFI = 0.95, TLI = 0.94, RMSEA = 0.05). The factor loadings are presented in Table 3 and range from 0.57 to 0.86. The omega reliability values for the latent factors ranged from 0.79 to 0.84, suggesting good internal consistency reliability. Although factors 1 and 2 are highly correlated (*r* = 0.84), they represent separate factors as indicated by the model difference in terms of χ^2^_(11)_ = 62.47, *p* < 0.001. We found that five latent factors were captured by the Values Inventory: Health/Wellbeing, Fundamental Human Values, Social Status, Personal Accomplishment/Global Advancement, and Community/Belonging. Health/Wellbeing included items such as “mental health”, “physical health”, and “sleep health”, Fundamental Human Values included items such as “happiness”, “open mindedness and family”, Social Status included items such as “social status”, “social influence” and “personal public image”, Personal Accomplishment/Global Advancement included items such as “autonomy,” “equality,” and “gaining knowledge”, and Community/Belonging included items such as “belonging” and “diversity.”

**Table 3:** Confirmatory factor analysis of the values inventory.

Items	Health/wellbeing	Fundamental human values	Social status	Personal accomplishment/global advancement	Community/ belonging
Mental health	0.80				
Physical health	0.75				
A comfortable life	0.72				
Self-care	0.70				
Sleep health	0.69				
Open mindedness		0.73			
Happiness		0.70			
Self-esteem		0.70			
Truthfulness		0.67			
Family		0.59			
Personal public image			0.86		
Social influence			0.81		
Social status			0.79		
Gaining knowledge				0.62	
Equality				0.61	
Autonomy				0.61	
Independence				0.60	
A balanced life				0.59	
Work ethic				0.59	
Freedom				0.57	
Belonging					0.76
Being liked					0.74
Diversity					0.69

Factor loading values calculated as Omega.

Table 4 presents the standardized regression coefficients from an SEM with the five latent factors, which were regressed on demographic variables. The estimates reflect the effect of each predictor on each latent factor, controlling for all the others in the model. Health/Wellbeing and Fundamental Human Values were rated higher among participants who were older. Higher income predicted higher ratings of Health/Wellbeing as well as greater ratings of Personal Accomplishment/Global Advancement. Education was a strong predictor of both Social Status and Community/Belonging, with higher levels of education corresponding to higher scores on these factors. Each additional dependent predicted lower factor ratings of Health/Wellbeing and Personal Accomplishment. Finally, males, older adults, and white participants each reported lower Social Status compared to other groups. These results highlight how different demographic characteristics relate to value orientations.

**Table 4:** Standardized effects of demographic variables on five values inventory latent factors from the SEM model.

Item	Health/wellbeing	Fundamental human values	Social status	Personal accomplishment/ global advancement	Community/ belonging
Age	**0.17** ^*^ **[0.06, 0.27]**	**0.23** ^**^ **[0.13, 0.34]**	−0.15^*^[−0.25, **−0.04]**	0.08 [−0.03, 0.19]	0.01 [−0.11, 0.12]
Sex, male	0.04 [−0.07, 0.15]	0.10 [−0.01, 0.20]	**−0.12** ^*^ **[−0.22, 0–.02]**	−0.02 [−0.13, 0.09]	0.03 [−0.09, 0.14]
Marital status, married	−0.08 [−0.21,.06]	−0.10 [−0.24, 0.04]	0.09 [−0.04, 0.22]	−0.05 [−0.20, 0.09]	0.03 [−0.11, 0.18]
Income	**0.22** ^*^ **[0.08, 0.36]**	0.13 [−0.01, 0.27]	0.14 [0.00, 0.27]	**0.22** ^*^ **[0.07, 0.37]**	0.12 [−0.03, 0.27]
Education	0.12 [−0.01, 0.25]	0.11 [−0.03, 0.24]	**0.24** ^**^ **[0.11, 0.36]**	0.13 [−0.01, 0.27]	**0.19** ^*^ **[0.04, 0.33]**
Dependents	−0.19^**^[−0.30, **−0.08]**	−0.11 [−0.25, 0.02]	−0.01 [−0.13, 0.12]	−0.16^*^[−0.29, **−0.03]**	−0.04 [−0.17, 0.10]
Race-ethnicity, white	0.04 [−0.07, 0.14]	0.11 [0.00, 0.22]	−0.11^*^[−0.21, **−0.01]**	0.00 [−0.12, 0.11]	0.06 [−0.04, 0.17]

Bold values indicate statistically significant results. Beta values [confidence internals].

^*^^*^p < 0.001.

^*^p < 0.05.

**Figure 1:**
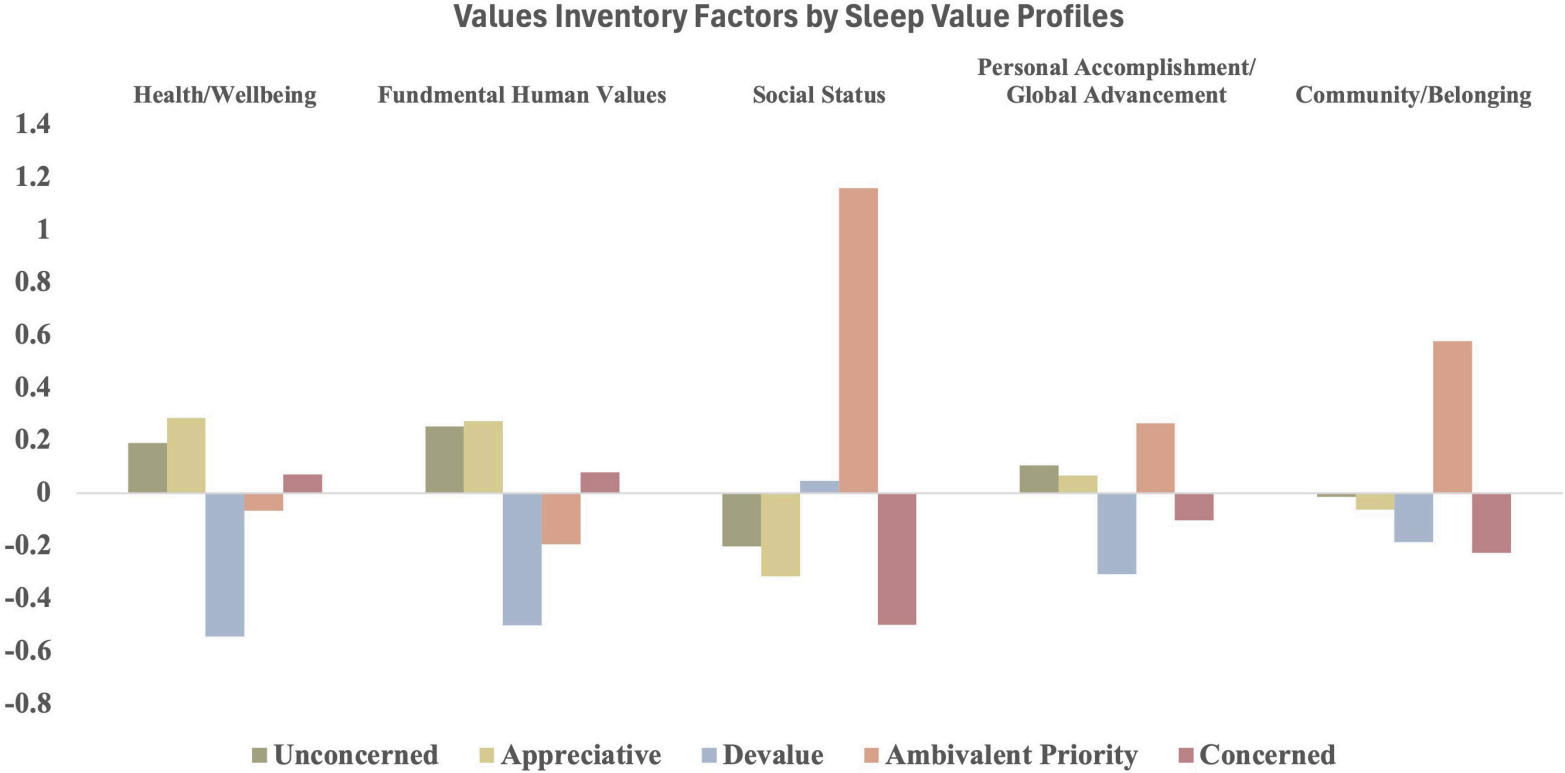
Values inventory factors by sleep value profiles mean differences.

The MANOVA revealed significant group differences across all factors (see Table 5, Figure 1). The Appreciative profile reported the highest endorsement of Health/Wellbeing and Fundamental Human Values, with scores significantly greater than those observed in the Unconcerned profile. In contrast, the Devalue profile consistently showed the lowest endorsement of Health/Wellbeing, Fundamental Human Values, and Personal Accomplishment/Global Achievement. The Ambivalent Priority profile placed strong emphasis on Social Status and Community/Belonging relative to the Unconcerned profile. The Concerned profile demonstrated lower valuation of Social Status alongside with a low valuation of Community/Belonging.

**Table 5:** Group comparison of sleep value profiles across values inventory factors.

Values Inventory factor	Unconcerned	Appreciative	Devalue	Ambivalent priority	Concerned
Health/wellbeing	–	**0.30 (0.08)** ^***^	–0.53 (0.08)^***^	−0.55 (0.1)	0.08 (0.1)
Fundamental human values	–	**0.29 (0.06)** ^***^	–0.48 (0.07)^***^	–0.18 (0.08)^*^	0.10 (0.1)
Social status	–	–0.35 (0.1)^***^	0.01 (0.1)	**1.1 (0.1)** ^***^	–0.54 (0.2)^**^
Personal accomplishment/global achievement	–	0.06 (0.06)	–0.31 (0.06)^***^	**0.26 (0.07)** ^***^	−0.11 (0.1)
Community/belonging	–	−0.08 (0.07)	**-0.21 (0.07)** ^**^	**0.56 (0.09)** ^***^	–0.25 (0.1)^*^

Bold values indicate statistically significant results. Unconcerned profile was used as the reference group.

^***^p < 0.001.

^**^p < 0.01.

^*^p < 0.05.

## Discussion

This study had three primary aims: to validate the Values Inventory and the placement of sleep health value within individuals' broader value systems, to examine how the resulting value factors relate to demographic variables and to evaluate how the Values Inventory corresponds with the Sleep Value Item Bank and its previously established sleep value profiles (Sherriff et al., 2025a). The EFMM analysis identified two classes, indicating that participants differed in how strongly the five Values Inventory factors were represented rather than reflecting entirely different structures. In both classes, five similar factors were present, but the strength and pattern of the factor loadings differed, suggesting that certain life values carry different levels of importance depending on an individual's broader value orientation. These results imply that life values operate within a shared structure but vary in emphasis across individuals, supporting the interpretation of the scale as capturing multiple dimensions that may present differently across individuals. The Values Inventory demonstrated strong psychometric properties, supporting a five-factor structure: Health/Wellbeing, Fundamental Human Values, Social Status, Personal Accomplishment/Global Advancement, and Community/Belonging, with high internal consistency and acceptable model fit. Sleep health, emerged as an important variable within the Health/Wellbeing factor, suggesting that participant responses view sleep as a pillar of their health as many sleep experts have advocated. In addition to this conceptual importance, we also observed meaningful demographic relationships with sleep value profiles.

Demographic analyses revealed that life values fluctuate meaningfully across demographics suggesting that sleep values are not merely static psychological traits but are nested dimensions of sleep health that shift in response to an individual's broader socioeconomic and social context. Applying the Socio-Ecological Model (Grandner, 2019), sleep value can be understood as a psychological dimension of sleep health that is shaped by the social and societal systems in which individuals participate. Our findings suggest that individual-level factors (beliefs and values) are nested within dynamic social-level factors. Older adults placed greater importance on Health/Wellbeing and Fundamental Human Values, while placing less emphasis on Social Status. This pattern suggests that as individuals age, their priorities shift toward intrinsic and health-related concerns rather than outward status markers, an individual-level change that occurs within the broader social context of evolving roles and work status Higher income was also associated with greater prioritization of Health/Wellbeing and Personal Accomplishment/Global Advancement, indicating that individuals with more financial resources may have greater capacity or opportunity to invest in health and achievement-oriented values. Education similarly predicted stronger endorsement of Social Status and Community/Belonging, reflecting how educational attainment often shapes both social positioning and the value placed on collective or civic engagement. Differences in income and education further demonstrate how broader social-level factors, particularly socioeconomic status, influence the extent to which individuals prioritize health-related values. Within the context of family structure, one of the most influential social-level factors, having more dependents was associated with lower prioritization of Health/Wellbeing and Personal Accomplishment. This pattern suggests that caregiving demands may limit individuals' ability to prioritize their own health or personal goals. This work highlights an important direction for future research examining how to mitigate the negative impact of social-level factors on individual-level dimensions of sleep health. Taken together, value orientations are closely tied to individuals' life contexts and socioeconomic systems, suggesting that interventions targeting health or sleep behaviors may benefit from being tailored beyond the individual to address them within a broader system.

Finally, the alignment between the Values Inventory factors and the sleep value profiles showed that the way people value sleep reflects broader personal and social priorities. These profiles represent distinct groups of people where the five previously determined factors of sleep value, wanting, prioritizing, devaluing, preferring, and appreciating, coexist in unique patterns within individuals. The multivariate analysis revealed clear differences across all factors. The Appreciative profile reflects an active enjoyment and favorable view of sleep, though sleep is viewed more as a valued aspect of life than as an urgent priority. Individuals in this profile showed the highest levels of Health/Wellbeing and Fundamental Human Values, suggesting that strong sleep valuation is part of a broader commitment to personal health and prosocial principles. The Devalue profile reflects individuals who do not want, appreciate, or prefer sleep and instead engage in sleep primarily out of necessity, often holding a generally negative attitude toward sleep. Individuals in this profile showed the lowest levels of Health/Wellbeing, Fundamental Human Values, Personal Accomplishment, and Global Advancement, suggesting that low sleep valuation may be part of a broader pattern of reduced personal and societal investment. The Ambivalent Priority profile reflects individuals who paradoxically both want and prioritize sleep but simultaneously devalue it, leading to internal conflict between desires, values, and behavior. Individuals in this profile showed higher valuation of Social Status and Community and Belonging, suggesting that inconsistent sleep prioritization may reflect competing social demands or concerns. The Concerned profile is characterized by high levels of wanting, appreciating, and prioritizing sleep, likely reflecting a response to insufficient or disrupted sleep, as individuals in this profile reported the highest levels of sleep disturbance and depression. This group also showed low valuation of Social Status, Community and Belonging, indicating a distinct pattern in which concern about sleep is not strongly tied to broader social values. The Unconcerned profile is characterized by individuals who neither strongly value nor devalue sleep and low levels of sleep disturbance. This group showed no strong pattern of values across the Values Inventory factors.

Understanding these value-based profiles may also have important implications for clinical practice. Identifying an individual's broader value orientation may help clinicians tailor sleep interventions more effectively. For example, individuals in the Concerned profile who place high value on Health and Wellbeing but struggle to attain the sleep they strongly value may benefit from cognitive restructuring or behavioral strategies that target maladaptive sleep-related beliefs and reduce worry. In contrast, individuals in the Unconcerned or Devalue profiles, who place less importance on Health and Wellbeing, may require interventions that increase motivation for sleep-promoting behaviors by linking sleep to other personally meaningful goals or life values. Considering these broader value systems may help clinicians better align sleep interventions with the motivational priorities of the individual. Overall, these findings suggest that sleep valuation is closely connected to broader value systems and highlight the potential importance of aligning sleep health interventions with individuals' personal values. Future research in this area may further clarify how personal value systems influence sleep-related attitudes and behaviors.

Our findings contribute to the broader literature of human values by including sleep value as a clear and measurable component within broader value systems. Foundational frameworks by Schwartz, Reiss, and Hofstede identify key dimensions of personal and societal values, yet none include sleep as an explicit element despite its central role in health and daily functioning (Hofstede, 1980; Reiss, 2004; Schwartz, 2012; Schwartz and Boehnke, 2004). Although sleep can conceptually intersect with constructs such as conservation, survival needs, or indulgence vs. restraint, these theories do not capture sleep value as a distinct contributor to decision making. The present study addresses this gap by validating the Values Inventory and demonstrating a clear five-factor structure that places sleep value within the Health and Well-being domain, which aligns with theorized conceptualizations of sleep health. This placement suggests that sleep is experienced not as an isolated preference but as part of a broader orientation toward maintaining physical and mental health. By showing that sleep value fits coherently into a well-supported value structure, our findings extend existing theories and highlight the need for value frameworks to include sleep as a meaningful part of what people prioritize in daily life.

The alignment between the Values Inventory factors and the sleep value profiles further clarifies how sleep value fits within individuals' socioecological context and larger value systems. The finding that the Appreciative sleep profile also valued the Health/Wellbeing factor the most out of the other profiles is consistent with previous research that gratitude and appreciation-based interventions are linked to a wide range of positive psychological and physiological outcomes. For example, Diniz et al. (2023)) found that gratitude interventions increased mental health and decreased symptoms of anxiety and depression in patients showing the tangible effects of appreciation. Moreover, Fagley (2018)) demonstrated that beyond gratitude, appreciation predicted positive affect on emotions independent of the Big Five Personality Traits (McCrae and Costa, 1987), meaning that appreciation effects well-being even outside of personality. This aligns with the broaden-and-build theory of positive emotion which maintains that positive emotions, such as joy, foster action (Fredrickson, 2004). Applied to appreciation, the theory might suggest that increasing appreciation could increase the desire to recognize value and prioritize, particularly, important aspects of life such as health and sleep. Therefore, the implications of this study go beyond promoting sleep prioritization and importance alone. Indeed, fostering appreciation for sleep may be the key driver of meaningful change in public priorities, attitudes, and valuation of sleep health. Together, these results show that sleep value is embedded in stable and meaningful aspects of the value system and that interventions aimed at improving sleep may be more effective if they align with or enhance individuals' broader value orientations, particularly appreciation for health and wellbeing.

Despite the strengths of this study, limitations are acknowledged. Participants were sampled from an online US population, limiting generalizability to subgroups and non-US populations. The absence of behavioral measures of sleep value limits interpretations of how values influence specific sleep and health outcomes and prevented us from determining the extent to which potential biases, such as social desirability, response fatigue, or individual differences in interpreting value-based or sleep-related items, may have influenced the results. Additionally, due to the cross-sectional nature of the study, we were unable to draw causal inferences from the observed response patterns. However, the use of a large sample strengthens confidence in the stability of the identified value structure, and the consistency of our findings with established theories of values and sleep supports the broader relevance of these patterns.

Future research can build on the present study by addressing these limitations and exploring new questions raised by this work. Longitudinal designs may help clarify how sleep valuation changes over time and in response to life events such as aging, parenthood, or work transitions. The use of stratified sampling could provide a clearer picture of how values interact with age, demographic characteristics, and life stages. Incorporating objective measures of sleep will be important to understanding individual value profiles correspond with real-world sleep behaviors and outcomes. Behavioral health metrics such as actigraphy-derived sleep timing and activity measures, as well as nutritional indicators, may allow researchers to better characterize how differing valuations of sleep influence health and well-being. For example, examining whether individuals who devalue sleep but still experience poor sleep report better well-being than those who highly value sleep but are unable to attain it. Such findings could suggest that the meaning individuals assign to sleep loss, rather than the behavior itself, moderate its psychological impact. Another important direction for future research is the use of culturally and socioeconomically diverse samples to examine whether ambivalent or devalued sleep profiles reflect socially structured value conflicts (e.g., sleep vs. productivity or social connection). Individuals exposed to early life adversity, discrimination, racism, microaggressions, or marginalization may develop enduring conflicts around rest, safety, and social belonging that shape how sleep is valued. Understanding these dynamics may help guide the development of more targeted interventions. For example, psychosocial strategies such as motivational interviewing or approaches that enhance sleep self-efficacy may improve sleep outcomes among individuals who experience value-based conflicts around sleep. Ultimately, understanding how individuals value sleep relative to other life values may offer important insight into the mechanisms underlying poor sleep health and inform more personalized approaches to sleep health promotion.

In conclusion, this study validated the Values Inventory as a reliable tool for understanding how life values relate to sleep and illuminated how sleep is embedded into the broader values people hold. While previous research has been conducted on value systems, the Values Inventory is the first to include sleep health specifically. Value systems are shaped by demographics such as age, education, and family demands, and these patterns influence both how sleep is perceived and potentially how it is prioritized. Future research can further explore these dynamics using more diverse samples and objective sleep measures to further explore the nuanced relationship between sleep values and behavior and help inform sleep intervention tailored to individual value orientations.

## Data Availability

The original contributions presented in the study are publicly available. This data can be found here: https://doi.org/10.33774/coe-2024-2l4zj.

## References

[B1] Buysse D. J. (2014). Sleep health: can we define it? Does it matter? Sleep 37, 9–17. doi: 10.5665/sleep.3298 24470692 PMC3902880

[B2] CDC (2016). 1 in 3 Adults Don't Get Enough Sleep [Online]. Atlanta, GA: Centers for Disease Control and Prevention. Available online at: https://archive.cdc.gov/www_cdc_gov/media/releases/2016/p0215-enough-sleep.html (Accessed July 1, 2025).

[B3] Diniz G. Korkes L. Tristão L. S. Pelegrini R. Bellodi P. L. Bernardo W. M. (2023). The effects of gratitude interventions: a systematic review and meta-analysis. Einstein 21:eRW0371. doi: 10.31744/einstein_journal/2023RW0371 37585888 PMC10393216

[B4] Fagley N. S. (2018). Appreciation (including gratitude) and affective well-being: appreciation predicts positive and negative affect above the big five personality factors and demographics. Sage Open 8:2158244018818621. doi: 10.1177/2158244018818621

[B5] Fredrickson B. L. (2004). The broaden–and–build theory of positive emotions. Philos. Trans. R. Soc. London. Ser B: Biol. Sci. 359, 1367–1377. doi: 10.1098/rstb.2004.1512 15347528 PMC1693418

[B6] Grandner M. A. (2019). “*Chapter 5 - Social-ecological model of sleep* health,” in Sleep and Health, ed. M. A. Grandner (Cambridge: Academic Press). doi: 10.1016/B978-0-12-815373-4.00005-8

[B7] Hedin G. Norell-Clarke A. Hagell P. Tønnesen H. Westergren A. Garmy P. (2020). Facilitators and barriers for a good night's sleep among adolescents. Front. Neurosci. 14:92. doi: 10.3389/fnins.2020.00092 32116531 PMC7019014

[B8] Hofstede G. (1980). Culture and organizations. Int. Stud. Manag. Organ. 10, 15–41. doi: 10.1080/00208825.1980.11656300

[B9] Huang Y.-C. Lin T.-T. Yang C.-M. (2023). Prioritization of sleep: discrepancies between attitude and practice. J. Sleep Med. 20, 160–168. doi: 10.13078/jsm.230019

[B10] Ignjatović S. Pavlović Z. Todosijević B. (2025). *Outliving oneself through the next* generations:(grand) parenthood and values in later life. Psychol. Aging 40, 294–307. doi: 10.1037/pag0000879 39846988

[B11] Kay D. B. Simmons Z. Nielson S. A. Braithwaite S. R. Esplin C. (2023). A first glimpse at the latent structure of sleep valuation using a sleep valuation item bank. Nat. Sci. Sleep 15, 127–137. doi: 10.2147/NSS.S386838 36974200 PMC10039622

[B12] Kline P. (2014). An Easy Guide to Factor Analysis. London: Routledge.

[B13] Knutson K. L. Van Cauter E. Zee P. Liu K. Lauderdale D. S. (2011). Cross-sectional associations between measures of sleep and markers of glucose metabolism among subjects with and without diabetes: the coronary artery risk development in young adults (CARDIA) sleep study. Diabetes Care 34, 1171–1176. doi: 10.2337/dc10-1962 21411507 PMC3114508

[B14] Laugsand L. E. Vatten L. J. Platou C. Janszky I. (2011). Insomnia and the risk of acute myocardial infarction: a population study. Circulation 124, 2073–2081. doi: 10.1161/CIRCULATIONAHA.111.025858 22025601

[B15] McCrae R. R. Costa P. T. (1987). Validation of the five-factor model of personality across instruments and observers. J. Pers. Soc. Psychol. 52:81. doi: 10.1037/0022-3514.52.1.81 3820081

[B16] McNeish D. (2018). Thanks coefficient alpha, we'll take it from here. Psychol. Meth. 23, 412–433. doi: 10.1037/met0000144 28557467

[B17] Muthén L. K. Muthén B. O. (1998-2023). Mplus User's Guide, 8th Edn. Los Angeles, CA: Muthén & Muthén.

[B18] Nielson S. A. Taylor J. Simmons Z. Decker A. N. Kay D. B. Cribbet M. R. (2021). Sleep valuation is associated with components of sleep health and daytime functioning in a college sample: a survey study. Int. J. Environ. Res. Public Heal. 18:5644. doi: 10.3390/ijerph18115644 PMC8197495 34070462

[B19] Petrofsky L. A. Heffernan C. M. Gregg B. T. Smith-Forbes E. V. Sturdivant R. X. (2023). Sleep and military leaders: examining the values, beliefs, and quality of sleep and the impact on occupational performance. Mil. Med. 189, 1023–1031. doi: 10.1093/milmed/usad040 36919969

[B20] Ravyts S. G. Dzierzewski J. M. (2024). Sleep disturbance, mental health symptoms, and quality of life: a structural equation model assessing aspects of caregiver burden. Clin. Gerontol. 47, 484–493. doi: 10.1080/07317115.2020.1783042 32597344 PMC7767889

[B21] Reiss S. (2004). Multifaceted nature of intrinsic motivation: the theory of 16 basic desires. Rev. Gen. Psychol. 8, 179–193. doi: 10.1037/1089-2680.8.3.179

[B22] Rokeach M. (1974). Change and stability in American value systems, 1968–1971. Public Opin. Q. 38, 222–238. doi: 10.1086/268153

[B23] Rokeach M. (1979). Understanding Human Values: Individual and Societal. Mumbai: Free Press.

[B24] Ruggiero A. R. Peach H. D. Gaultney J. F. (2019). Association of sleep attitudes with sleep hygiene, duration, and quality: a survey exploration of the moderating effect of age, gender, race, and perceived socioeconomic status. Heal. Psychol. Behav. Med. 7, 19–44. doi: 10.1080/21642850.2019.1567343 PMC8114360 34040837

[B25] Russo C. Danioni F. Zagrean I. Barni D. (2022). Changing personal values through value-manipulation tasks: a systematic literature review based on Schwartz's theory of basic human values. Eur. J. Invest. Heal. Psychol. Educ. 12, 692–715. doi: 10.3390/ejihpe12070052 PMC9319275 35877452

[B26] Schwartz S. H. (2012). An overview of the Schwartz theory of basic values. Online Read. Psychol. Cult. 2:11. doi: 10.9707/2307-0919.1116

[B27] Schwartz S. H. Boehnke K. (2004). Evaluating the structure of human values with confirmatory factor analysis. J. Res. Pers. 38, 230–255. doi: 10.1016/S0092-6566(03)00069-2

[B28] Sherriff D. Solomon S. Ward L. Nielson S. A. Yang C. Duraccio K. M. . (2025a). Exploring sleep value: distinct profiles and individual differences. Res. Direct. Sleep Psychol. 2:e3. doi: 10.1017/slp.2024.7

[B29] Sherriff D. Ward L. Calvin D. Klingonsmith B. Kay D. B. (2025b). Sleep value and sleep resilience are important dimensions of sleep health and we measured them: methods for the sleep resilience and variance in sleep valuation (SRVIV) study. Res. Direct. Sleep Psychol. 2:e1. doi: 10.1017/slp.2024.6

[B30] Sherriff D. Yang C. Duraccio K. M. Kay D. B. (2026). Sleep resilience is a novel dimension of sleep health that is associated with sleep-related impairment: a confirmatory factor analysis, internal consistency, and predictive validity assessment of the Sleep Resilience Questionnaire in a US adult sample. Sleep zsag029. doi: 10.1093/sleep/zsag029 41631604

[B31] Sorscher A. J. (2008). How is your sleep: a neglected topic for health care screening. J. Am. Board Fam. Med. 21, 141–148. doi: 10.3122/jabfm.2008.02.070167 18343862

[B32] Vgontzas A. N. Liao D. Bixler E. O. Chrousos G. P. Vela-Bueno A. (2009). Insomnia with objective short sleep duration is associated with a high risk for hypertension. Sleep 32, 491–497. doi: 10.1093/sleep/32.4.491 19413143 PMC2663863

[B33] Wells M. E. Vaughn B. V. (2012). Poor sleep challenging the health of a nation. Neurodiagn. J. 52, 233–249. doi: 10.1080/21646821.2012.11079859 23019761

